# Prognostic value of immunoexpression of CCR4, CCR5, CCR7 and CXCR4 in squamous cell carcinoma of tongue and floor of the mouth 

**DOI:** 10.4317/medoral.22904

**Published:** 2019-04-24

**Authors:** Catherine-Bueno Domingueti, João-Baptista-Macuco Janini, Lívia-Máris-Ribeiro Paranaíba, Carlo Lozano-Burgos, Pablo Olivero, Wilfredo-Alejandro González-Arriagada

**Affiliations:** 1 University José do Rosário Vellano, Biomedicina of Varginha, Brazil; 2 Postgraduate Program in Biological Sciences, Federal University of Alfenas, Brazil; 3 Institute of Diagnostic and Prevention (IPD Laboratory), Varginha, Brazil; 4 Hospital Carlos Van Buren, Valparaíso, Chile; 5 Centro de Investigación Interoperativo en Ciencias Odontológicas y Médicas (CICOM), Universidad de Valparaíso, Valparaíso, Chile; 6 Facultad de Medicina, Universidad de Valparaíso, Valparaíso, Chile; 7 Patología y Diagnóstico Oral, Facultad de Odontología, Universidad de Valparaíso, Valparaíso, Chile

## Abstract

**Background:**

Diverse studies have evidenced that chemokines can play a critical role in pathogenesis of oral squamous cell carcinoma (SCC). The main chemokines involved in oral carcinogenesis, tumor invasion and metastasis are CCR4, CCR5, CCR7 and CXCR4, and our aim was to evaluate the prognostic value of the immunoexpression of these chemokines in SCC of tongue and floor of the mouth.

**Material and Methods:**

A retrospective descriptive study of the immunohistochemical expression of CCR4, CCR5, CCR7 and CXCR4 in paraffin-embedded samples of 124 patients with SCC of the tongue and floor of the mouth was performed, considering 98 cases from Brazil and 26 cases from Chile. Associations between variables were analyzed using chi-square test. Survival curves were performed using the Kaplan-Meier method and compared with long-rank test. For multivariate survival analysis, the Cox hazard model was established. The level of significance established was *p*≤0.05.

**Results:**

The statistical analysis showed that samples with well or moderate WHO model differentiation (*p*=0.001) and a high expression of CCR5 (*p*=0.05) were significantly associated with a higher disease specific survival, which were also observed in Cox´s multivariate analysis (*p*=0.01). A higher expression of CCR7 (*p*=0.01) interfered significantly in disease-free survival in univariate analysis and in Cox´s multivariate analysis (*p*=0.05).

**Conclusions:**

These results support additional evidence, showing that chemokine receptors CCR5 and CCR7 are helpful as biomarkers of poor prognosis in patients with SCC of the tongue and floor of the mouth.

** Key words:**Oral squamous cell carcinoma, prognosis, survival, chemokine receptor.

## Introduction

Squamous cell carcinoma (SCC) is a malignant neoplasm, accounting for more than 90% of malignancies in the oral cavity (1). It is more frequent in men after the fifth decade of life, but interestingly, recent epidemiological studies have suggested that 4 to 6% of oral cancer cases in the world have occurred in young adults, aged 18-45 years (2). The etiology is multifactorial, with extrinsic and intrinsic factors contributing to the development of this disorder. The most correlated envvironmental factor is tobacco smoking and exposure of the mucosa to alcohol (3). The human papillomavirus (HPV), is especially associated with oropharyngeal carcinoma and its association with oral cavity cancer in not conclusive (4).

The treatment of these patients is based on surgery, radiotherapy and/or chemotherapy, that can include neck dissection when lymphadenopathy is evidenced, but occult cervical metastasis can occur (5). Despite the advances in research and treatment options, the prognosis of patients with oral SCC has remained practically static in the last decades, remaining between 50 and 60% for a period of 5 years (6). This low survival rate is mainly due to late diagnosis, local invasion and high propensity for regional and distant dissemination (7).

For many years, research in carcinogenesis and cancer progression focused on tumor cells, especially genetic and epigenetic alterations. However, it has been revealed that cancer is a more complex disease, involving strong interactions between cells of the tumoral microenvironment (TM) and others components of extracellular matrix (ECM), including carcinoma-associated fibroblasts (CAFs), immune and inflammatory, and supporting blood and lymphatic endothelial cells (8).

Chemokines are small chemotactic cytokines that play a key role in tumor progression, migration, leukocyte activation, angiongenesis and metastasis. Some studies report that cytokines may have different effects on the tumor, being able to keep the phenotype invasive (9). These molecules act by selective membrane, that are linked to seven G-protein-coupled receptors (GPCRs) that present two main families, CCR and CXCR (10). Chemokines are classified in four groups, according to conservation and spacing of cysteines in CXC, CC, C and CX3C (11). Have been reported that CXCR4 mediate growing signals and promote metastasis (10), CCR5 acts in recruitment of effector cells and antigen-presenting cells (macrophages) (12), CCR4 and CCR7 has been associated to the ability of neoplastic cells to promote lymph node metastasis. CCR7 can also promote the proliferation of neoplastic cells, adhesion, migration, invasion and angiogenesis in oral tumorigenesis (9).

Chemokine receptors play a key role in the development, progression and metastasis of cancer, and were reported in various types of cancer, as kidney (13), ovary (14), and head and neck SCC (9,15). Our group previously reported the immunohistochemical expression of CCR1, CCR3, CCR4, CCR5, CCR7 and CXCR4 in SCC of the head and neck (oral cavity, oropharynx and larynx), suggesting an important role of CCR5 and CCR7 in cancer progression (15). Diverse studies reported that chemokines can also play a critical role in pathogenesis and progression of oral SCC (9,12,15). Based on these arguments, the aim of the current study was to evaluate the prognostic value of the immunohistochemical expression of CCR4, CCR5, CCR7 and CXCR4 related to clinicopathological parameters in samples of SCC of tongue and floor of the mouth.

## Material and Methods

-Patients and sample collection

Herein we are reporting a retrospective descriptive study of the immunohistochemical expression of CCR4, CCR5, CCR7 and CXCR4 in paraffin-embedded tissues of 98 cases from Hospital do Bom Pastor (Varginha-MG, Brazil) and 26 cases from Hospital Carlos van Buren (Valparaíso, Chile), between 1998 and 2014. Clinicopathological data was collected from patients’ files. All histopathological slides were revised and the clinical stage was re-diagnosed according to the Eighth edition of the American Joint Committee on Cancer (AJCC) Staging Manual, Head and Neck Section (16).

Inclusion criteria chosen for this study were: 1- patients with a diagnosis of OSCC of tongue and floor of the mouth, and treated at this hospital, 2- surgical treatment performed according to standard procedure and consisting of resection of the primary tumor, 3- clinicppathological data and samples of paraffin-embedded tumor for evaluation. The blocks were kindly provided by IPD Laboratory (Institute of Diagnostic and Prevention) of Varginha, Brazil, responsible for anatomopathological analysis of all samples of the Hospital Bom Pastor, and the Anatomic Pathology Service of the Hospital Carlos van Buren of Valparaíso, Chile.

The parameters obtained in the medical charts of each patient were: age, gender, smoking and drinking habit, tumor localization (tongue or floor of month), clinical stage, treatment (surgery, surgery associated with radiotherapy or surgery associated with radiotherapy and chemotherapy), margins status, lymph node involvement, perineural invasion, lymphocytic response and recurrence/ metastasis (local, lymph node, distance or not). The exclusion criteria considered poor information or insufficient tissue for immunohistochemical reactions.

The study was approved by the Ethics Committee in Research of the, Federal University of Alfenas (protocol number: 1.775.304) and University of Valparaíso, Chile (protocol number: CB 051-14).

-Immunohistochemical staining and analysis

Histological sections at 3μm were diaphanized in three sequences Histological Clearing Agent, Histo-Clear (National Diagnostic), hydrated in three decreasing concentrations of alcohol and distilled water. The antigen retrieval procedure, was performed using EnVision™ Flex Target Retrieval Solution (Dako) at 95 °C for 20 min, followed by endogenous enzyme block (EnVision FLEX peroxidase-blocking reagent) for 5 min, and the sections were incubated with primary antibodies CCR4 (rabbit, polyclonal antibody PA532698, dilution 1:200, Invitrogen Inc., USA), CCR5 (rabbit, polyclonal antibody PA529011, dilution 1:500, Invitrogen Inc., USA), CCR7 (rabbit, polyclonal antibody PA533401, dilution 1:150, Invitrogen Inc., USA) and CXCR4 (rabbit, polyclonal antibody PA3305, dilution 1:1000, Invitrogen Inc., USA), overnight at 4ºC. The next day, EnVisionTM Flex Mouse Linker was added for 15 min (excluding cases with primary antibody CXCR4), and EnVision FLEX / HRP for 30 min, the revelation was incubation with diaminobenzidene (DAB) chromogen, the slides were counterstained with hematoxylin.

**Figure 1:**
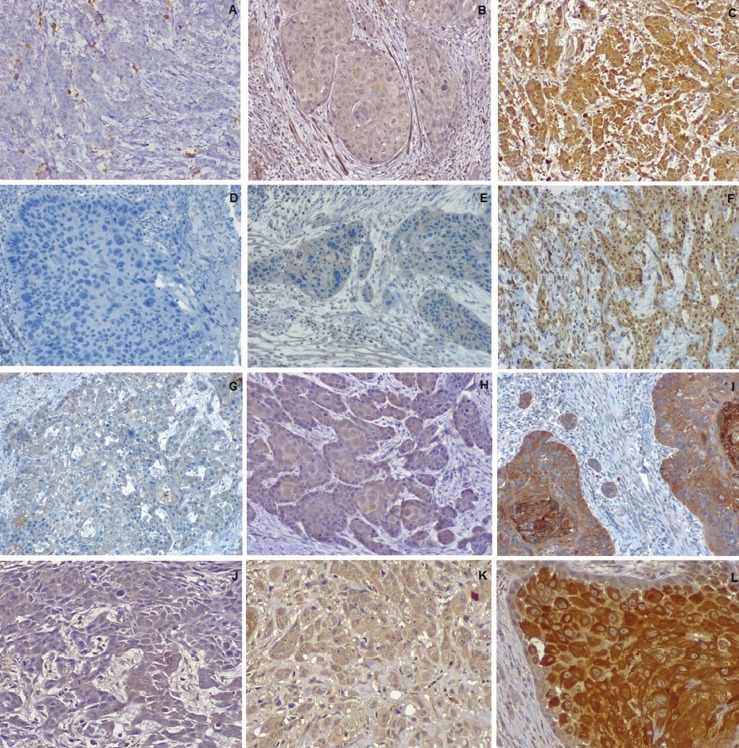
Low/negative, moderate and high staining intensity for CCR4 (A,B,C), CCR5 (D,E,F), CCR7 (G,H,I) and CXCR4 (J,K,L) (20x objective).

The immunohistochemical analysis was applied with a semiquantitative scoring system according to a previously published method. This visual method was performed by two observers at the same time and defined two evaluation criteria (Fig. 1):

• Percentage of positive cells with, 0 (negative), 1 (1%–25%), 2 (26%–50%), 3 (51%–75%) and 4 (76%–100%) 

• Stain intensity with, 0 (negative), 1 (weak), 2 (moderate) and 3 (intensive staining).

The final score was obtained by multiplying the percentage of positive cells with the stain intensity score, and was classified as low expression (score 0-3) and high expression (score 4-12) (15).

-Statistical analysis

The level of significance established was *p*≤0.05. Curves for analysis of disease-specific survival and disease-free survival were using the Kaplan-Meier method and compared with long-rank test, for multivariate survival analysis, the Cox proportional hazard model with a stepwise method was established. Associations between clinicopathological parameters and immunohistochemical expression of CCR4, CCR5, CCR7 and CXCR4 using the chi-square test were also analyzed.

## Results

The clinicopathological features of the studied population is summarized in  Table 1,  Table 1 continue. Eighty-two patients (66.1%) were men, with a median-age of 62 years-old. Eighty-three (66.9%) patients were smokers and 61 (49.2%) reported alcohol consumption. Regarding T stage, 68 patients (54.8%) were classified as T1/T2 and 52 patients (41.9%) as T3/T4. In the N stage, the highest prevalence was N0 with 68 patients (54.8%), followed by 24 patients (19.3%) with N1, and 22 patients (17.7%) with N2. Forty-four patients (35.5%) were classified as clinical stage I or II (early stage) and 76 (61.3%) were classified at advanced clinical stage (stages III or IV). Four patients did not present the TNM stage in their medical records. Surgery as exclusive therapy was performed in 24 patients (19.3%), whereas 43 (34.7%) were treated by combination of surgery and radiotherapy and 52 (41.9%) had the combination of surgery, radiotherapy and chemotherapy ( Table 1,  Table 1 continue).

**Table 1:**
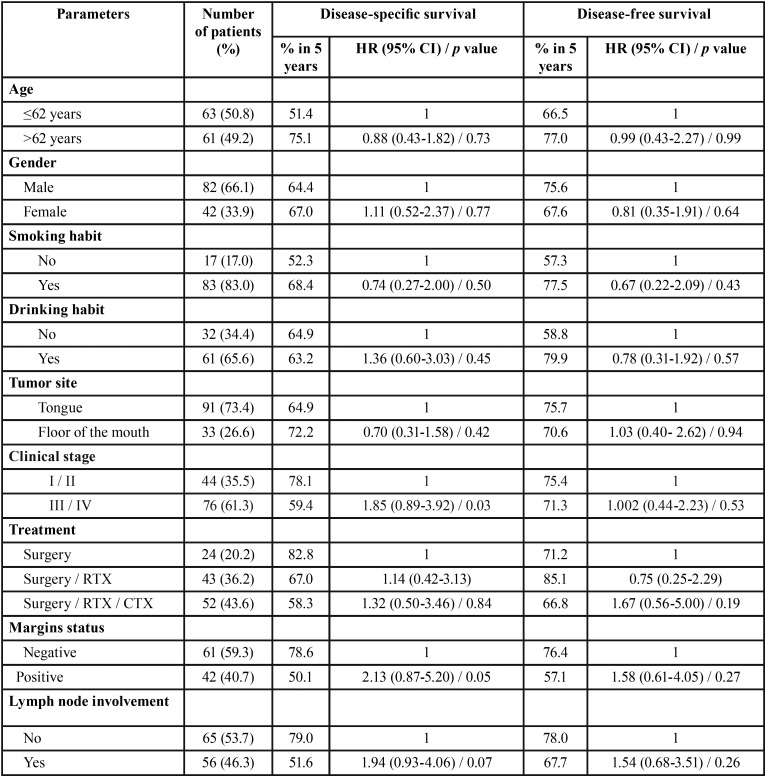
Clinical and pathological features and univariate analysis for disease-specific survival and disease-free survival of patients with squamous cell carcinoma of tongue and floor of the mouth.

**Table 1 continue:**
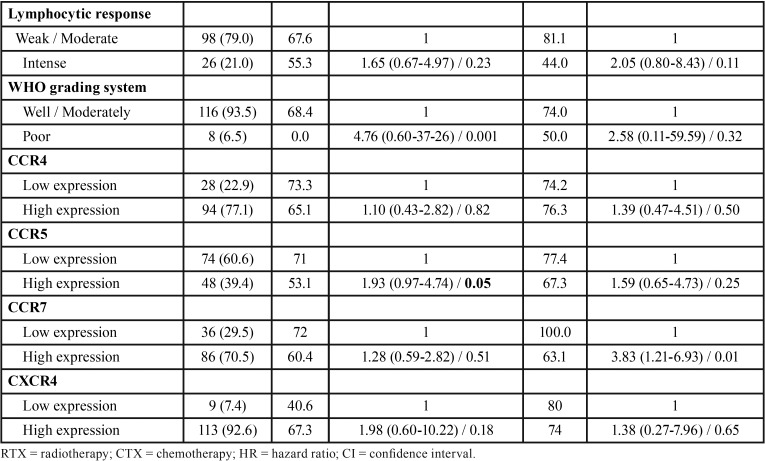
Clinical and pathological features and univariate analysis for disease-specific survival and disease-free survival of patients with squamous cell carcinoma of tongue and floor of the mouth.

The analysis of immunohistochemical markers showed that 39.4% (n=48) of cases had a high expression for CCR5, 69.3% (n=86) for CCR7, 75.8% (n=94) for CCR4, and 91.1% (n=113) for CXCR4. Most surgical pieces presented negative margins (59.3%, n=61) and perineural invasion (53.2%, n=66). From 124 patients evaluated, 91 (73.4%) received cervical resection. The results revealed that 93.5% of cases (n=116) were classified as well/moderate differentiated according to WHO classification system ( Table 1,  Table 1 continue). The overall survival ranged from 1 to 136 months, with a mean of 90 months for disease-specific survival and 91 months for disease-free survival. Six patients were excluded from the evaluation of survival analysis because the lack of information in their medical records.

In the univariate analysis, was observed that clinical stage (*p*=0.03), positive surgical margins (*p*=0.05), poor differentiation (*p*=0.001) and CCR5 expression (*p*=0.05) revealed significance for specific survival of disease and a high expression of CCR7 (*p*=0.01) interfered with disease-free survival ( Table 1,  Table 1 continue). However, in the Cox’s multivariate analysis, poor differentiation (*p*=0.01) is significant for specific survival ( Table 2) and high expression of CCR7 (*p*=0.05) for free-disease survival ( Table 3).

**Table 2:**
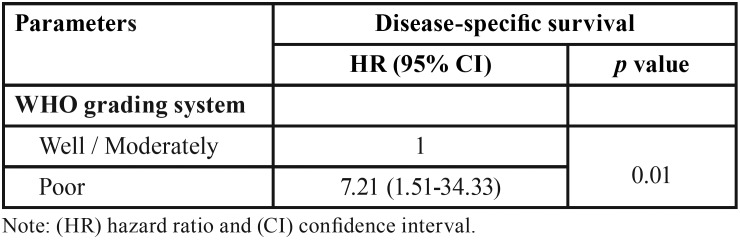
Multivariate Cox analysis for disease-specific survival in patients with squamous cell carcinoma of tongue and floor of the mouth.

**Table 3:**
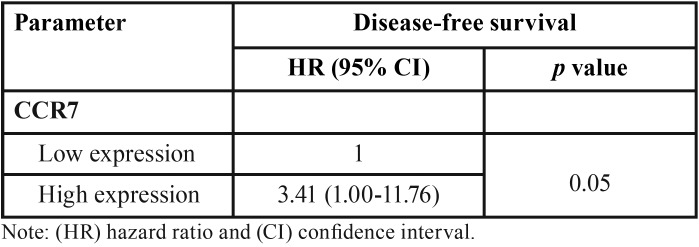
Multivariate Cox analysis for disease-free survival in patients with squamous cell carcinoma of tongue and floor of the mouth.

Association between immunohistochemical expression of CCRs and clinicopathological features are showed in  Table 4,  Table 4 continue. A significant association between the immunoexpression of CCR4 and positive margins (*p*=0.01) was observed. For CCR5, was identified that clinical staging (*p*=0.05) and intense lymohocytic response (*p*=0.01) were significant. CCR7 showed significance with gender (*p*=0.02) and recurrence (*p*=0.05). CXCR4 was expressed in a high number of tumors, mainly in the center of islands, with a less intense expression in the borders of islands, for this reason it did not show any association with clinicopathological parameters.

**Table 4:**
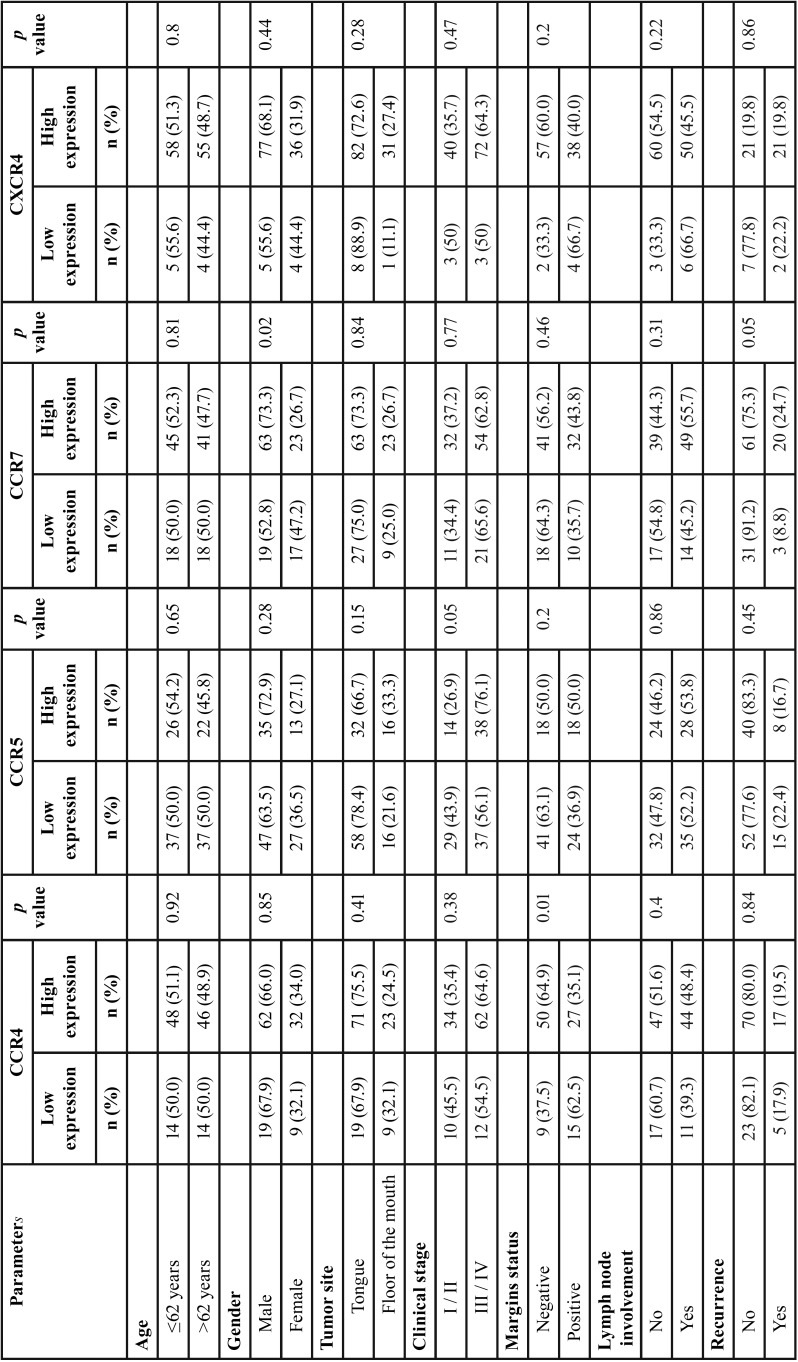
Statistical analysis chi-square method of clinical and pathological parameters with immunoexpression of CCR4, CCR5, CCR7 and CXCR4.

**Table 4:**
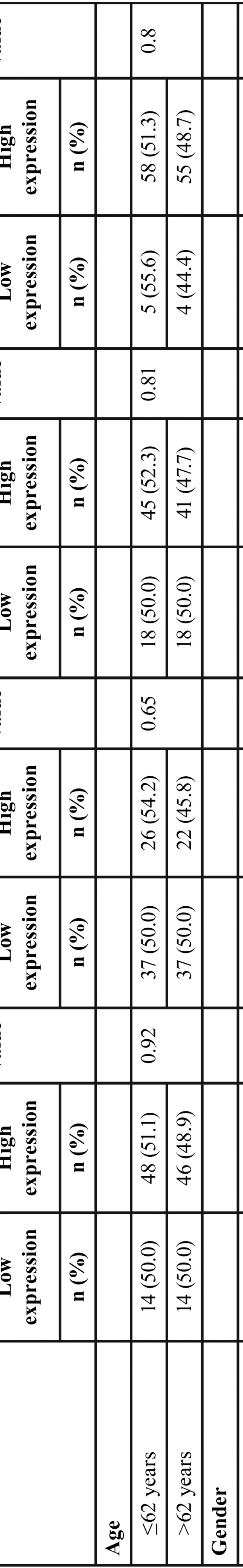
Statistical analysis chi-square method of clinical and pathological parameters with immunoexpression of CCR4, CCR5, CCR7 and CXCR4.

## Discussion

SCC is a malignant neoplasia of epithelial origin that represents more than 95% of malignant tumors of oral cavity, and accounts for almost 4% of malignancies of humans (17,18). It is frequently diagnosed in advanced stage, and in our sample 61.3% (n=76) of patients were diagnosed in advanced stage (III or IV), which is associated with an elevated propensity for local invasion, regional and distant dissemination. The identification of potential new prognostic markers could help to determinate an accurate prognosis for oral SCC.

The traditional histopathologic WHO gradation system showed positive association in univariate analysis for disease specific survival (*p*=0.001). Despite these results, many studies report that the application of WHO classification to predict the prognosis in patients with SCC have been criticized because the absence of important features related to tumorigenesis, such as tumor thickness, vascular invasion, margins evaluation and regional lymph nodes) (18), in addition to the subjectivity in the sample analysis and the weak correlation with the response to the therapy (16,19).

The current study revealed that the immunohistochemical expression of CCR5 showed significance for disease specific survival in univariate analysis (*p*=0.05), and when it was compared with clinicopathological parameters, was observed significance with clinical stage (*p*=0.05) and lymphocytic response (*p*=0.01). Some studies demonstrated that CCR5 is an unfavorable marker in patients with cancer of breast (20), colon (21), pancreas (22) and melanoma (23). CCR 5 has two ligands, CCL3 and CCL5, and recently was reported that the axis composed by CCR5 and associated chemokines has pro-tumorigenic effect, playing an important role in oral cancer progression (24).

Some studies reveal that T cells have a crucial role in the modulation of antitumoral immune response. De Oliveira *et al.* demonstrated, that T cell migration to the tumor microenvironment is mediated by CCR5, promoting the SCC growing, through inhibition of antitumoral cells. Other study reveals that migration and death of oral tumor cells mediated by T cells, have the participation of CCR5, suggesting a new approach through modulation of CCR5 signals in monocytes and macrophages (12). Previous research reported that monocytes of patients with oral SCC present significantly reduced levels of CCR5 and reduction of migration when are compared with healthy patients. Migration of leukocytes is fundamental for the antitumoral activity of monocytes and macrophages, and this reduction can facilitate the suppression of the immunological system of patients with oral SCC (26). Gonzalez-Arriagada *et al.*, reported in samples of head and neck SCC, that CCR5 is associated to advanced stage, lymph node metastasis and lower survival. The current data show that patients with SCC of tongue and floor of the mouth, with a higher expression of CCR5, are associated with advanced clinical stage and worse prognosis. Recently was reported that the CCR5 antagonists reduce tumor growth and progression of colon cancer cells (27). For these reasons, we suggest that CCR5 is a chemokine that can permit a therapeutical approach to the treatment of SCC of tongue and floor of the mouth.

**Figure 2:**
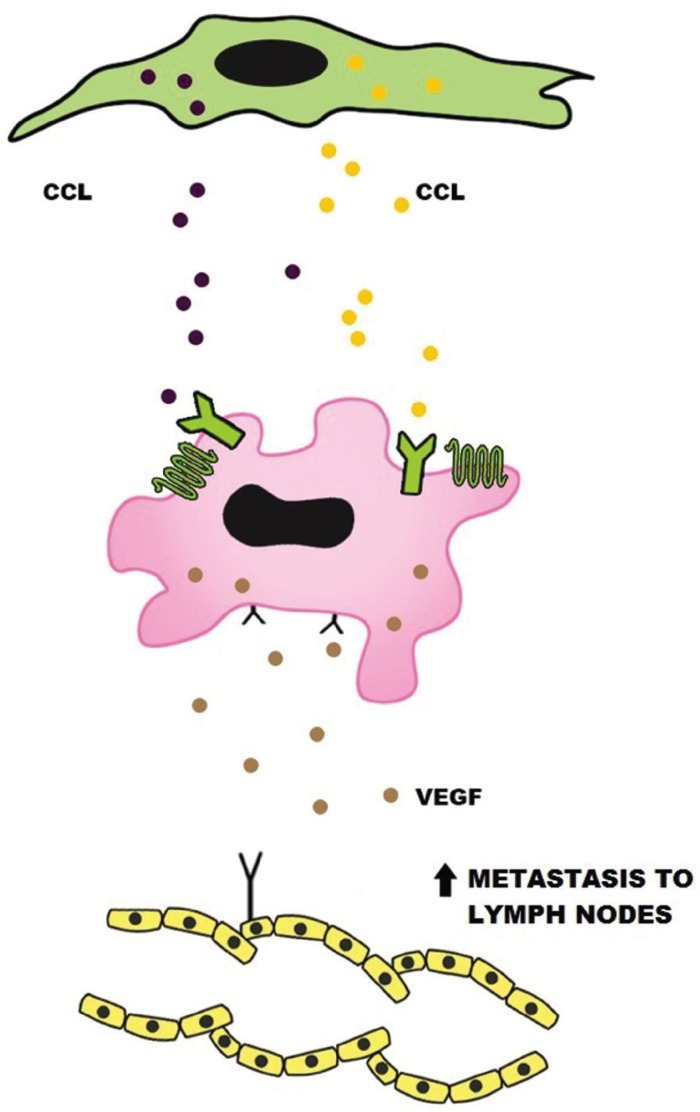
CCRs has chemokines as ligands. Chemokines are released by lymphoid CAFs, promoting lymphangiogenesis and migration of CCR+ neoplastic cells to lymph nodes.

Metastasis is a mechanism that depends of the migration through the extracellular matrix, adhesion to the vascular endothelium, invasion of blood vessels, extravasation and growing in a secondary organ (28). CCR7 have two ligands, CCL19 and CCL21. CCL19 is expressed in lymphoid tissues (28) and it can promote cellular migration and adhesion, favoring the metastasis (Fig. 2).

Additionally, we observed that the high expression of CCR7, presented significance for disease-free survival in univariate analysis (*p*=0.01) and Cox’s multivariate analysis (*p*=0.05). CCR7 also showed correlation with clinicopathological parameters, such as genre (*p*=0.02) and recurrence/metastasis (*p*=0.05). Retrospective studies about diverse neoplasias showed that tumor cells that express CCR7 are present in cancer of breast (29), colorectal (30) and pancreas (31).

It was reported in tongue SCC that the high immunohistochemical expression of vascular endothelial growth factor C (VEGF-C), vascular endothelial growth factor receptor 3 (VEGFR-3), CCR7 and semaphorin 3E (SEMA3E) are predictors of metastasis. It was demonstrated that these factors can be useful to evaluate metastasis in lymph nodes of SCC, with the aim to improve the oral SCC patients’ survival after treatment (32). Previous research reported that CCR7 regulate metastasis in head and neck SCC (28,33,34,35). The importance of the signaling way Janus activated kinase-3 (Jak3) in the metastasis of malignant head and neck tumors mediated by CCR7 and its ligands, can be a new target for treatment of these patients (36). Also, was reported that CCR7 can activate JAK2/STAT3 and to promote metastasis. In this way, CCR7/JAK2/STAT3 regulate metastasis by E-cadherin mediated epithelial-mesenchymal transition (EMT) (33). EMT represents a biologic process that allows biochemical, molecular and morphological modifications in a polarized epithelial cell, that normally interacts with basal membrane. These modifications result in the acquisition of a mesenchymal cell phenotype, with the capacity of migration, invasion and resistance to apoptosis (37). The role of CCR7 immunoexpression to predict cervical lymph node metastasis of oral SCC has been previously reported (38), so our results confirm the predictive utility of this marker in oral cancer.

Recently, CCR7 was associated with recurrence, gender, smoking habit and bad prognosis in head and neck cancer (15). Our results demonstrated that patients with SCC of tongue and floor of the mouth and a high expression of CCR7 are associated with gender and recurrence/metastasis. In this way, CCR7 can allow that SCC cells of tongue and floor of the mouth become more invasive and pro-metastatic, suggesting a therapeutic approach of these patients.

## Conclusions

Finally, our results show that CCR7 and CCR5 can be helpful as prognostic markers and as a therapeutic approach of patients with SCC of tongue and floor of the mouth. The association of CCR5 and CCR7 chemokine/chemokine receptor axis with poor prognosis in oral SCC needs future molecular research to study mechanisms that lead to tumor growth and progression, considering that immunohistochemical studies can only confirm statistical relationship.

We suggest the name oncochemotaxis for the mechanism that lead the malignant cells to invasion and migration to the lymph node through the expression of chemokine receptors in its surface attracted by chemokines.
